# Microfluidic EBG Sensor Based on Phase-Shift Method Realized Using 3D Printing Technology

**DOI:** 10.3390/s17040892

**Published:** 2017-04-18

**Authors:** Vasa Radonić, Slobodan Birgermajer, Goran Kitić

**Affiliations:** BioSense Institute—Research Institute for Information Technologies in Biosystems, Dr Zorana Đinđića 1a, 21000 Novi Sad, Serbia; b.sloba@gmail.com (S.B.); gkitic@gmail.com (G.K.)

**Keywords:** microstrip sensor, electromagnetic band gap (EBG), microfluidics, 3D printing, fused deposition modelling (FDM), phase-shift method

## Abstract

In this article, we propose a novel microfluidic microstrip electromagnetic band gap (EBG) sensor realized using cost-effective 3D printing technology. Microstrip sensor allows monitoring of the fluid properties flowing in the microchannel embedded between the microstrip line and ground plane. The sensor’s operating principle is based on the phase-shift method, which allows the characterization at a single operating frequency of 6 GHz. The defected electromagnetic band gap (EBG) structure is realized as a pattern in the microstrip ground plane to improve sensor sensitivity. The designed microfluidic channel is fabricated using a fused deposition modelling (FDM) 3D printing process without additional supporting layers, while the conductive layers are realized using sticky aluminium tape. The measurement results show that the change of permittivity of the fluid in the microfluidic channel from 1 to 80 results in the phase-shift difference of almost 90°. The potential application is demonstrated through the implementation of a proposed sensor for the detection of toluene concentration in toluene–methanol mixture where various concentrations of toluene were analysed.

## 1. Introduction

Microfluidics is a technology of manipulating the small quantity of fluids in the range of microliters to picoliters in one or a network of microchannels. Since microfluidic technology allows for operation and control of fluids on a sub-microscale, it found the applications in various scientific and engineering disciplines such as inkjet printing, chemistry, environment, biomedicine, etc. [1,2,3,4,5]. Nowadays, advanced microfluidic biochips integrate into a single chip a number of operations such as sample pre-treatment and preparation, cell separation, and transport, mixing and/or separation of fluids together with micromechanical, optical, and electronic components for sensing and detection.

In order to integrate advance functions into a singular chip, modern microfluidics combine multiple technologies including microelectromechanical systems technology, injection moulding, photolithography and X-ray lithography, laser ablation and micromachining, etc. [1,4,5]. However, all stated microfabrication techniques are relatively complex, time-consuming processes that typically require additional manual manufacturing procedures.

Nowadays, 3D printing technology attracts significant attention due to its low-cost, simple fabrication process that can be realized in a single run, good system compatibility, and presence of a number of different materials with good optical, biocompatible, chemical or mechanical properties [6,7,8]. The 3D printing offers the opportunity to fabricate the whole microfluidic device in a single run without the need for additional assembly processes. A wide range of biomaterials, such as living cells and growth factors, could also be directly printed using 3D printing technology [8].

The 3D printing technology is based on different manufacturing methods, such as stereolithography, multi-jet modelling, electron beam melting, bioprint, and fused deposition modelling (FDM). The most commercially available 3D printers operate according to the FDM method, which has relatively low accuracy and speed in comparison to other methods. FDM 3D printers build structure layer-by-layer from the bottom up by extruding thermoplastic filament through a heated nozzle and deposit it in fine threads along the extrusion path. Recently, FDM technique has been employed to produce LEDs, sensors, antennas, and electrodes within biological tissue [6,7].

Fabrication of microfluidic channels by FDM is still a challenge because of several limitations: extruded filaments cannot be arbitrarily joined at intersections, the lack of structural integrity between the layers results in weak seals, and the size of the extruded filament is larger than a typical channel used in microfluidics. So far, the utilisation of FDM 3D printing technology for applications in different microfluidic devices has been reported in a number of publications in literature [6,7,8,9,10,11,12,13,14,15]. A reactor with fluidic 3 mm tube was fabricated using acrylonitrile butadiene styrene (ABS) polymer in [9], but this device suffers from leakage and low operation pressure. Microfluidic channel of 800 μm was used in the realization of organic and inorganic synthesis reactionware for electrochemical and spectroscopic analysis [10]. Furthermore, fluidic devices with the same size of the microchannel were used for nanoparticle preparation and electrochemical sensing [11], and detection of cancer protein biomarkers based on supercapacitor-powered immunoarray [12]. However, it is still challenging to fabricate leak-proof microfluidic channels narrower than 800 µm with the help of commercially available filaments using FDM 3D printing technology. In [13], a custom FDM printer was designed for enhanced resolution of the microchannel, where 350 μm wide polycaprolactone microchannels have been applied in the realization of 3D nervous-system-on-a-chip.

Recent studies on microfluidics have been expanded to fluid detection at radio frequency (RF) and microwave frequencies [14,15,16,17,18,19,20]. RF and microwave microfluidics use fluids as a substitutive dielectric material for microwave antennas [14,15], transmission lines [16], or resonators [17]. In that manner, the characteristic parameters of the fluids were determined based on measured impedance [15,16], resonant frequency [17,18,19], insertion loss, or the phase of the signal that propagates along the transmission line [20]. For the sensing applications, the most promising method is the method based on the phase measurement of the propagating signal [20,21], since it has a relatively fast response, allows for characterization at single frequency, and it is the least sensitive to insertion loss.

In this paper, we propose a novel microfluidic microstrip sensor realized using cost-effective 3D printing technology. The 350 µm wide microfluidic channel embedded in the microstrip substrate has been fabricated by conventional FDM 3D printing technology using polylactic acid (PLA) filament without any supporting layer or soluble material. Leak-proof structure has been ensured through careful optimization of the 3D model, infill factor and high infill/perimeter overlap settings. The proposed sensor is designed to allow monitoring of the dielectric properties of the fluid that flows in the microfluidic channel embedded between the microstrip line and ground plane realized using defected electromagnetic band gap (EBG) structure. A concept to improve microstrip sensor sensitivity based on defected EBG structure was proposed in [21], where it is demonstrated that the sensor sensitivity can be increased by reducing the wave group velocity using periodic patterns in the ground plane, which exhibits EBG effect [22]. The characteristics of different fluids in the microfluidic channel are analysed by the phase-shift measurements of the transmitted signal.

The potential application is demonstrated through the realization of the sensor for detection of toluene concentration in toluene–methanol mixture. Toluene is an aromatic hydrocarbon solvent and has numerous commercial and industrial applications, such as solvent in paints, thinners and glues. It is also used for printing and leather tanning processes, for disproportionation to a mixture of benzene, methanol and xylene, and in the production of a number of synthetic drugs. Toluene, methanol and their mixtures appear in a liquid state at room temperature. However, not only their liquids but also their vapour concentrations in the air can become extremely high and can have negative effects on work environments or can easily burn or explode [23,24]. Hence, the existence of a low-cost, high-sensitive sensor that is able to detect the exact concentrations of toluene or methanol in their mixtures is essential in their operations, storage or transportation. The advantage of the proposed design has been demonstrated through comparison between the fabricated sensor and other recently published microfluidic sensors which operate according to the phase-shift method.

## 2. Sensor Design

**Figure 1:**
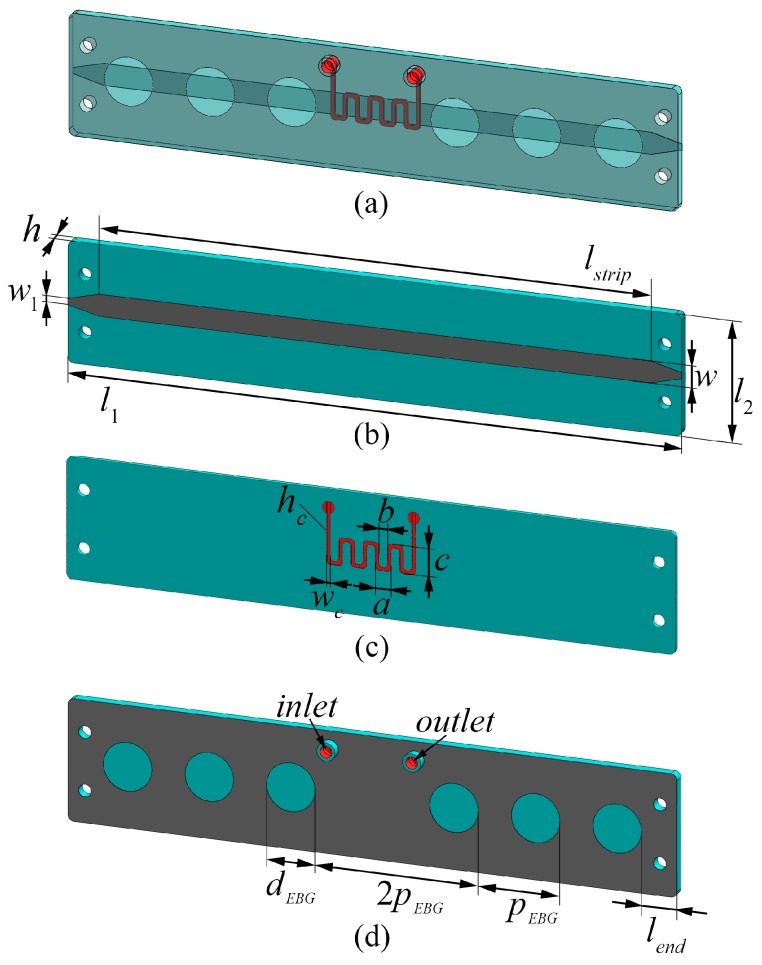
The layout of the proposed microfluidic electromagnetic band gap (EBG) sensor: (**a**) 3D view; (**b**) top layer; (**c**) substrate with embedded microfluidic channel; (**d**) bottom layer with defected EBG structure.

The 3D layout of the proposed microfluidic microstrip sensor with defected EBG etched in the ground plane is shown in Figure 1a. The designed sensor consists of three main parts: a microstrip line in the top layer, a microfluidic channel in the middle, and ground plane patterned with defected EBG structure in the bottom layer. The top layer, shown in Figure 1b, consists of 50 Ω microstrip line with length *L_strip_* and width *w*, respectively, and two tapers. To avoid the short circuit between end-launched sub-miniature A (SMA Southwest Microwave 292-04A-5) connectors’ ground and the microstrip line, two tapers were designed according to the manufacturer’s recommendation. The microfluidic channel bent in the shape of meander is embedded in the microstrip substrate above the defect in the EBG, i.e., the most sensitive location in the design, Figure 1c. Parameters *a*, *b* and *c* denote the dimensions of meandered microchannel, while *w_c_* and *h_c_* are the width and height of the channel, respectively. The total thickness of the substrate with embedded microfluidic channel is denoted as *h.* The bottom layer of the sensor, shown in Figure 1d, represents the ground plane realized using defected EBG structure, periodical structure that consists of etched holes with diameter *d_EBG_* placed at distance *p_EBG_*. The defect in EBG is realized under the microchannel by removal of the one periodic element. Introduction of the defected EBG structure improves sensitivity of the sensor in comparison to the conventional microstrip line. In addition to EBG, two holes that serve as inlet and outlet of the channel were designed in the bottom layer for assembly of microfluidic equipment to inject fluids into the channel, Figure 1d.

**Figure 2:**
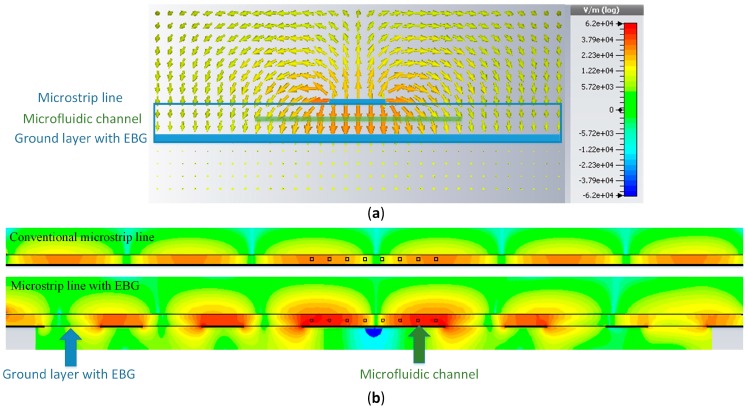
Electric field distribution of the proposed microfluidic EBG sensor: (**a**) orthogonal cross-section view; (**b**) comparison of the intensity of the electrical field along the microstrip line for conventional microstrip line and microstrip line with EBG.

Figure 2 shows the electrical filed distribution in the orthogonal cross section of the proposed sensor and comparison of the intensity of the electrical field along the microstrip line. The strongest electric field exists in the substrate between the microstrip line and the ground plane, i.e., the zone where the microfluidic channel is located. Moreover, in comparison with a conventional microstrip line, the intensity of the electric field is stronger in the vicinity of a defect in the EBG. Therefore, the changes of the dielectric constant of the liquid that flows in the channel will have the highest impact to the sensor response. In order to further increase the sensitivity of the sensor, microfluidic channel is bent in the shape of the meander without changing the cross-section dimensions of the channel. In that manner, the effective area of the fluid exposed to the strongest electrical field increases compared to the topologies that use straight microfluidic channel.

## 3. Sensor Operating Principle: Phase-Shift Method

Phase-shift method is based on a measurement of phase delay of the sinusoidal signal that propagates along the transmission line. The phase shift of the signal is determined by velocity and frequency of the signal as well as the physical properties of the transmission line:

(1) $$\Delta \varphi =\frac{\omega \;L_{TL}}{v_{p}},$$

where *ω* denotes angular velocity, *v_p_* is the phase velocity of the signal, and *L_TL_* is the transmission line effective length. On the other hand, the phase velocity of the signal is dependent on the properties of the surrounding medium of the transmission line. In general, the phase velocity of the signal can be determined as:

(2) $$v_{p}=\frac{\sqrt{2}}{\sqrt{\mu \varepsilon }}\frac{1}{\sqrt{1+\sqrt{1+\frac{\sigma^{2}}{\omega^{2}\varepsilon^{2}}}}},$$

where *μ* is magnetic permeability, *ε* is dielectric permittivity and *σ* is electric conductivity of the medium that surrounds the transmission line, [25]. If the operating frequency is high enough, the influence of conductivity can be neglected and expression for phase velocity can be reduced to:

(3) $$v_{p}=\frac{1}{\sqrt{\mu \varepsilon }},$$

which exclusively depends on permittivity and permeability of surrounding medium. In this way, cross-sensitivity with respect to conductivity can be avoided. This is particularly interesting if only the change in permittivity needs to be monitored, as in the case of detecting pollutants in water [26].

**Figure 3:**
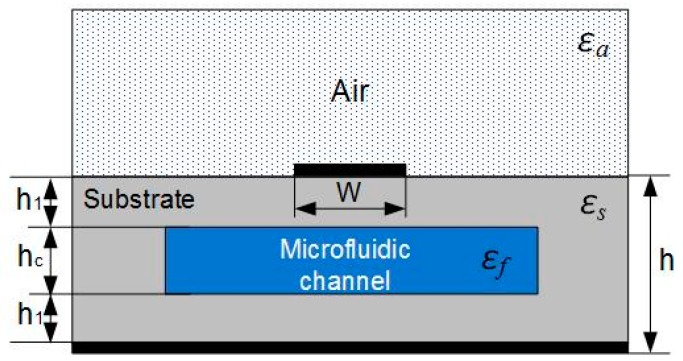
Cross section of the proposed sensor.

The proposed sensor is based on a transmission line in the form of microstrip architecture with degeneration in the ground plane and inhomogeneous surrounding medium, Figure 3. The surrounding medium of the microstrip line consists of the air from top and the microfluidic channel embedded in 3D printed substrate.

In order to apply the above-stated equations, the concept of effective permittivity can be introduced. In the simple case of the microstrip line, the effective permittivity can be approximated as:

(4) $$\varepsilon_{eff}=\frac{\varepsilon_{a}+\varepsilon_{sf}}{2}+\frac{\varepsilon_{a}-\varepsilon_{sf}}{2}\frac{1}{\sqrt{1+12\frac{h}{w}}}\;,$$

where *ε_a_* and *ε_sf_* are permittivities of air and combination of 3D printed substrate and fluid in the microfluidic channel, respectively [27].

The effective permittivity of the combination of the inhomogeneous dielectric substrate can be calculated using an equation for effective dielectric permittivity of the multilayered substrate, [28]:

(5) $$\varepsilon_{sf}=\frac{\left|d_{1}\right|+\left|d_{2}\right|+\left|d_{3}\right|}{\left|\frac{d_{1}}{\varepsilon_{s}}\right|+\left|\frac{d_{2}}{\varepsilon_{s+f}}\right|+\left|\frac{d_{3}}{\varepsilon_{s}}\right|}\;,$$

where the coefficients *d_n_* are:

(6) $$d_{1}=\frac{1}{\pi }\ln\left(2\frac{1+\sqrt{\frac{1}{\cosh\left(\frac{\pi w}{4h_{1}}\right)}}}{1-\sqrt{\frac{1}{\cosh\left(\frac{\pi w}{4h_{1}}\right)}}}\right),\;d_{2}=\frac{1}{\pi }\ln\left(2\frac{1+\sqrt{\frac{1}{\cosh\left(\frac{\pi w}{4\left(h_{1}+h_{c}\right)}\right)}}}{1-\sqrt{\frac{1}{\cosh\left(\frac{\pi w}{4\left(h_{1}+h_{c}\right)}\right)}}}\right),\;d_{3}=\frac{1}{\pi }\ln\left(2\frac{1+\sqrt{\frac{1}{\cosh\left(\frac{\pi w}{4\left(2h_{1}+h_{c}\right)}\right)}}}{1-\sqrt{\frac{1}{\cosh\left(\frac{\pi w}{4\left(2h_{1}+h_{c}\right)}\right)}}}\right).$$

The *ε_s+f_* is the dielectric constant of the middle layer with microchannel that can be calculated using Bruggeman formalism [29]:

(7) $$\varepsilon_{s+f}=V\varepsilon_{s}+\left(1-V\right)\varepsilon_{f}\;,$$

where *V* is the volumetric fraction of the microfluidic channel in the surrounding PLA substrate.

Based on the above equations, the operating principle of the sensor can be described. The change of the fluid’s properties in the microfluidic channel causes the change of *ε_s+f_*, which results in the change of effective permittivity of the microstrip. Consequently, the phase velocity changes, which alters the phase shift. It can be concluded that different values of the fluid’s permittivity is related to different phase shifts, which is a necessary condition for constructing a calibration curve.

The defected EBG pattern in the sensor ground plane is used to improve the sensitivity of the microstrip sensor. The introduction of the uniform EBG structure in the ground plane forms a frequency region where propagation is forbidden, i.e., bandgap in the transmission characteristic [22]. The group velocity that can be determined by the slopes of the bands of the propagation modes goes to zero at the bandgap edges in the case of the microstrip EBG sensors [21]. In that manner, for a constant operating frequency, a significant change in the group velocity can be observed in the case of the microstrip with EBG in comparison to the conventional microstrip line. A large decrease in the group velocity corresponds to the slow-wave effect.

The defect in the EBG results in a resonance in the bandgap, which frequency is determined by the size of the defect. By introducing defected EBG structure in the microstrip ground plane, the phase change significantly increases, especially at the frequencies that are close to the bandgap edges and at the resonance in the bandgap. As is shown in [21], at a constant frequency, the change in wave vector, *k* is larger for the band with lower group velocity (*Δk*). The phase change (*Δφ*) for a given change in permittivity is proportional to *Δk L_TL_*, where *L_TL_* is the effective length of the microstrip sensor. This is the main cause of increased phase change and therefore sensitivity of the proposed sensor.

It can be mentioned that from the phase difference measurement, the real part of rgw dielectric constant can be directly determined. The complex permittivity of the fluid can be obtained by measuring both the amplitude and the phase of the transmitted signal of the sensor and incident signal, or it can be reconstructed from Kramers–Kronig dispersion relations.

The phase-shift method allows characterization of a sample on single frequency, unlike resonant methods that require characterization over a range of frequencies. In addition, phase-shift measurement is less prone to the noise and less sensitive to the insertion loss. In that manner, this method is suitable for sensing of the high-loss materials.

## 4. Simulation Results

The characteristics of the proposed sensor, the influence of different geometrical parameters, optimization and the influence of the different fluids in the microchannel have been analysed using CST Microwave studio. PLA is chosen as substrate material since it is one of the most used thermoplastics in 3D printing. Initially, dialectic constant of the used 3D printed PLA material printed with 100% infill was determined to be 2.7 with tan*δ* equal to 0.01 at the frequency of 6 GHz. The optimized dimensions of the microstrip line, microfluidic channel and EBG structure have been determined to be: *h* = 1.5 mm, *l_strip_* = 89.84 mm, *w*_1_ = 1 mm, *w* = 3.6 mm, *l*_2_ = 20 mm, *l*_1_ = 100 mm, *h_c_* = 0.4 mm, *w_c_* = 350 µm, *a* = 2.35 mm, *b* = 1.65 mm, *c* = 4.1 mm, *d_EBG_* = 8 mm, *p_EBG_* = 13.4 mm, and *l_end_* = 5.8 mm.

**Figure 4:**
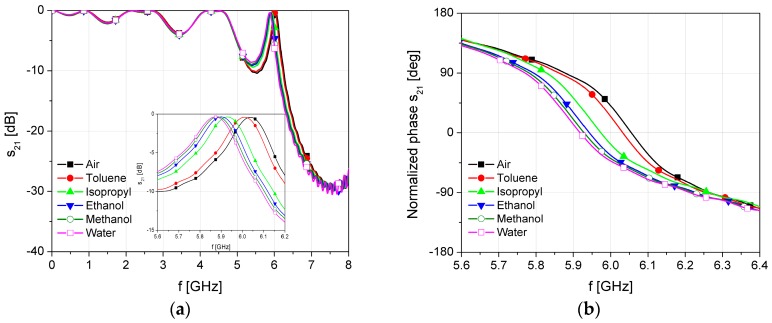
Simulation results of the proposed sensor with different fluids placed in the microchannel: (**a**) transmission characteristic; (**b**) normalized phase.

The simulation results of the proposed sensor with different fluids placed in the microchannel are shown in Figure 4. Each fluid in the simulation is modelled with its material parameters, i.e., its permittivity and dissipation factor, as shown in Table 1 [30].

The EBG structure is designed to provide bandgap between 5 and 9 GHz, while the defect in the EBG causes the resonant effect at 6 GHz. From the transmission characteristic, it can be seen that the resonant frequency of the defect in the band gap slightly shifts due to the change of the dielectric constant of the material in microchannel, Figure 4a. On the other hand, the phase change is increasing due to a decrease in the wave phase velocity, Figure 4b. The effect of the EBG structure is predominant at the frequency of 6 GHz where the wave phase velocity is minimal. This frequency is recognized as a frequency of interest since the change of the phase difference is the highest in that case. It should be noted that changes of the resonance and loss in the transmitted signal depend on the material in the microfluidic channel. However, the insertion losses do not fall below—10 dB in the worst case at operating frequency. Therefore, the phase of the transmitted signal can be measured using standard phase comparators or detectors that determine phase difference as a subtraction of the phase shift of the sensor transmitted signal and the phase of the excitation signal.

The simulation results show that the change of the fluid permittivity from 1 (air) to 80.1 (water) causes the phase-shift difference of 84°. Compared to the phase shift of the conventional microstrip line without defected EBG which is only 10.2° at 6 GHz, the proposed design shows eight times higher phase shift.

## 5. Fabrication and Measurement

Microfluidic channel, embedded into the substrate, was designed using the 3D modelling CAD software. The effects that occur during 3D printing, such as shrinkage and variations of the final dimensions, were taken into account in the final model to obtain designed dimensions of the channel and thickness of the substrate. 3D model has been imported into KISSlicer software to create the G-code with the following slicing parameters: layer thickness of 0.1 mm, extrusion width of 0.25 mm, number of perimeter equal to 4, infill factor of 100%, and the print speed of 20 mm/s. In order to make a leak-proof structure, the 3D printed model is designed using full infill factor and high infill/perimeter overlapping settings.

**Figure 5:**
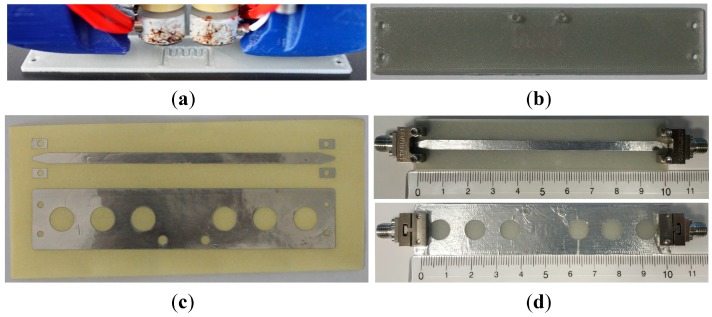
Sensor fabrication process: (**a**) Printing process of the 3D substrate with embedded microchannel; (**b**) Layout of the 3D printed substrate with embedded microfluidic channel; (**c**) Conductive top and bottom layers precisely cut with laser, and (**d**) Layout of the proposed sensors, top layer and bottom layer with mounted SMA connectors.

The microfluidic sensor’s substrate and microfluidic channel are printed simultaneously using a Felix 3.1 3D printer based on FDM technology. Biodegradable PLA thermoplastic filament with a diameter of 1.75 mm is used since it results in well-defined and quality printed structures. The extruder temperature was set to 190 °C for the first layer, while 185 °C was used for other layers. It should be noted that the designed microchannel is printed without any supporting material. Figure 5a shows the printing process of the 3D substrate with embedded microchannel.

**Figure 6:**
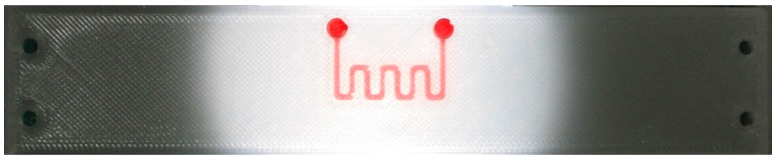
Layout of the 3D printed substrate with embedded microfluidic channel filled with coloured fluid.

**Figure 7:**
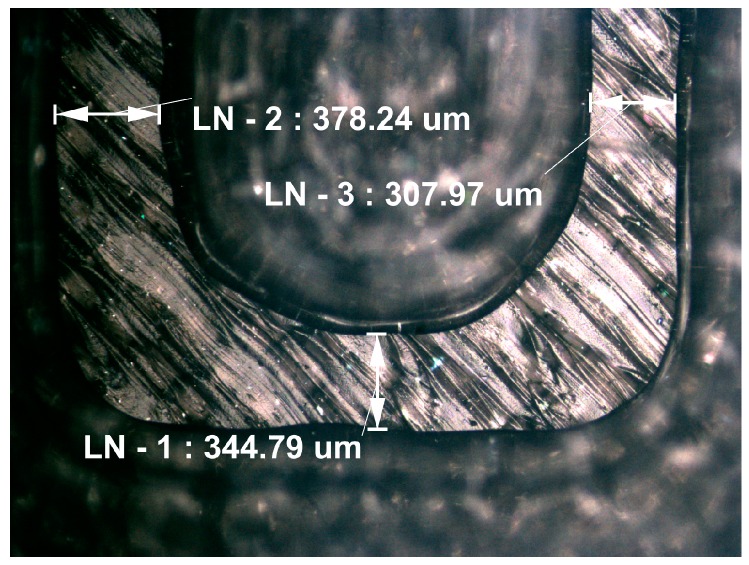
Cross section of the 3D printed microfluidic channel with measured widths.

The layout of the fabricated substrate and microfluidic channel filled with coloured fluid are shown in Figure 5b and Figure 6, respectively. The final dimensions of the fabricated microfluidic channel are determined by measuring the horizontal and vertical cross sections of the fabricated prototypes using the Huvitz HRM 300 profilometer, as shown in Figure 7. The measured dimensions of the fabricated microfluidic channel and substrate are: *h* = 1.52 mm, *l*_1_ = 100.1 mm, *h_c_* = 367–383 µm, *w_c_* = 343–384 µm, *a* = 2.15–2.71 mm, *b* = 1.45–1.72 mm, *c* = 4.02–4.23 mm. Imperfections of the 3D printing process mostly affect the dimensions of the channel causing the variation up to 10% from the designed values.

The conductive parts in the top and bottom layers were realized using 40 µm thick conductive aluminium sticky tape precisely cut with Rofin-Sinar PowerLine D laser, Figure 5c. The sticky conductive tape is accurately positioned and affixed from both sides of the substrate. The final layout of the proposed sensors with mounted end-lunch SMA connectors is shown in Figure 5d.

**Figure 8:**
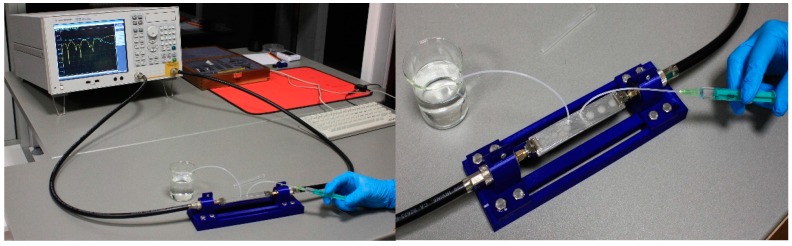
Measurement setup of the proposed sensor.

The measurement setup is shown in Figure 8, where the fluids injected are true additional tubules into the microfluidic channel using syringe. The characteristic parameters of the fabricated sensor were measured in the frequency range between 100 kHz and 8 GHz using two ports Agilent 8501C Vector Network Analyser.

**Figure 9:**
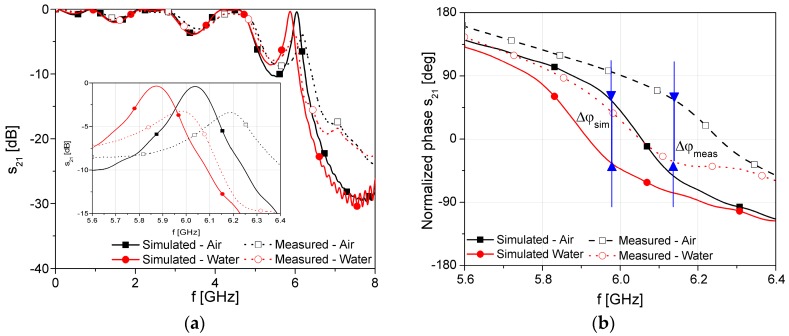
Comparison of the measured and simulated responses: (**a**) Transmission characteristic; and (**b**) Normalized phase.

The simulation and the measurement results are compared in terms of transmitted amplitude and phase, Figure 9. For better visibility, the measurements and simulations were compared only for two fluids, i.e., fluids which have the lowest and the highest dielectric constant, i.e., air and water. It can be seen that the simulated and measured results are in excellent agreement. The frequency where the phase shift shows the highest changes is slightly shifted to 6.15 GHz due to imperfection of the fabrication process, while the phase difference is slightly increased. On the other hand, the insertion losses do not fall below −10 dB in the worst case at the operating frequency.

**Figure 10:**
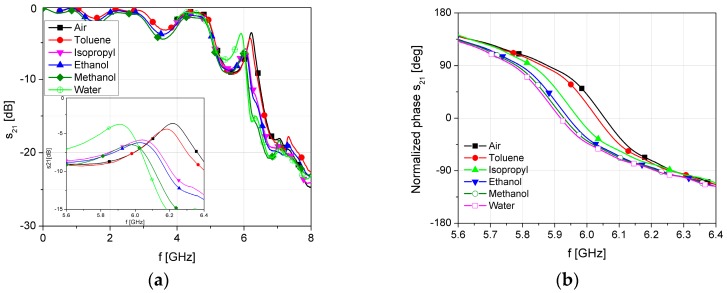
Measured results of the proposed sensor with different fluids inside the microfluidic channel: (**a**) Transmission characteristic; and (**b**) Normalized phase.

Figure 10 shows the measured transmission and phase characteristics of the proposed sensor with different fluids in the microfluidic channel in the frequency range of interest. The empty air channel is used as a reference value. For better visibility, the phase characteristic is normalized in the range from −180° to 180°.

For more comprehensive analysis, Table 2 summarizes the phase shifts for different fluids in the microfluidic channel obtained by simulations and measurements, as well as the phase shifts calculated based on the above-stated equations, where *ε_sf_* denotes effective permittivity of a combination of 3D printed substrate and fluid in the microfluidic channel, *ε_eff_* is the total effective permittivity, while *Δφ_cal_, Δφ_sim_* and *Δφ_meas_* are calculated, simulated and measured phase shifts, respectively. The calculated and simulated results agree well with the measured results.

**Figure 11:**
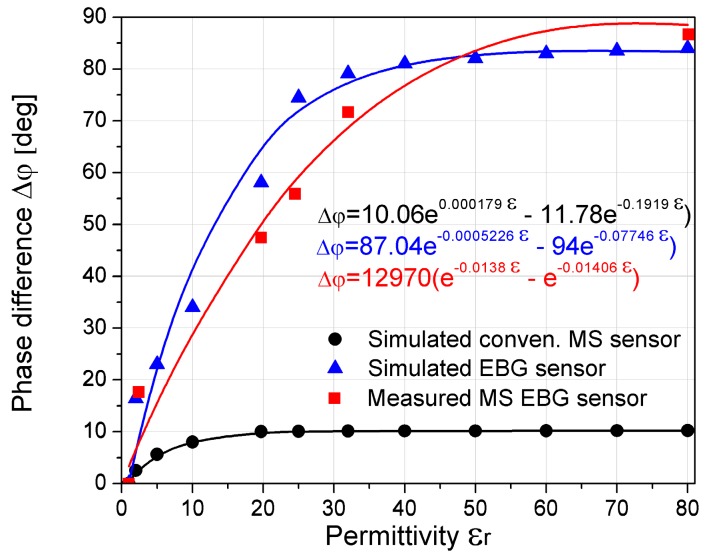
Sensitivity of the proposed EBG sensor compared to the conventional microstrip line sensor (dots) and the corresponding fitting curves (lines) with equations.

The sensitivity of the proposed sensor can be defined as a ratio of the phase difference of sensor response with fluid and air in the microchannel, divided by permittivity of used fluid. Figure 11 shows the phase-shift change of the proposed microfluidic EBG sensor with respect to the change of permittivity of the fluid. For a better comparison, the results of the conventional microstrip line sensor, as well as results obtained from simulations, are added in Figure 11. The exponential fitting curves and corresponding equations that provide excellent curve-fitting are also presented in Figure 11. The Curve Fitting Tool in the Matlab was used. It can be seen that the proposed sensor shows relatively high and almost linear dependence for the fluid materials with permittivity lower than 30. For the higher values of the permittivity, the change in phase is relatively small and sensor goes to saturation. It can be seen that in the case of the conventional microstrip line, eight times lower sensitivity is achieved while the saturation occurs for permittivity of 20. Furthermore, the measurement results show the better linearity in comparison with the simulations, especially for the lower values of dielectric constant.

**Figure 12:**
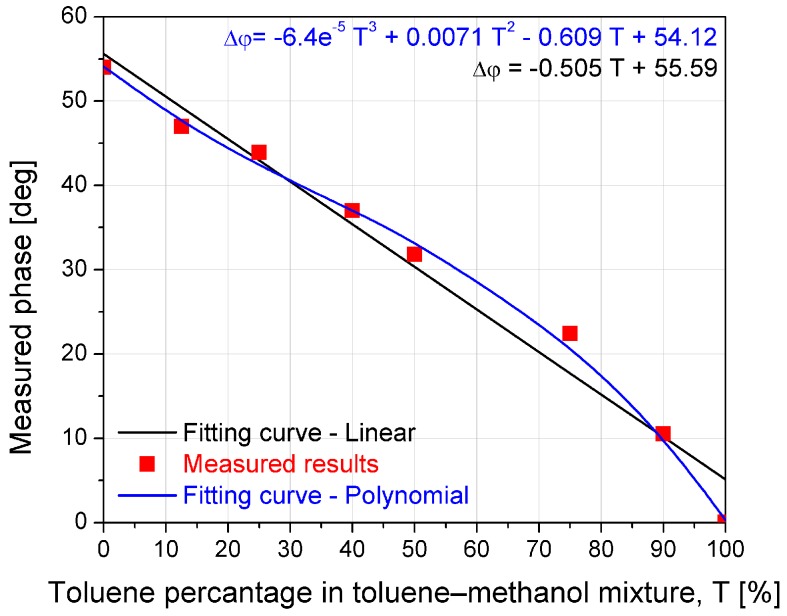
Measurement of the toluene concentration in toluene–methanol mixture: sensor measured response (dots) and the corresponding fitting curves (lines) with equations.

The potential application is demonstrated through the implementation of proposed sensors for the detection of toluene concentration in toluene–methanol mixture, where various concentrations of toluene were analysed. Figure 12 illustrates the measured phase difference when the toluene concentration varies from 0% to 100%. The linear and polynomial fitting curves and corresponding equations that better describe the measured phase dependence on the concentration of toluene in the toluene–methanol mixture are also shown in Figure 12. The experimental results show the phase difference changes for 54 degrees when the concentration of toluene is changed from 0% to 100%. In addition, the proposed sensor shows almost linear shift with sensitivity of 0.540° per percentage of toluene. These results successfully demonstrate and confirm the application of the proposed microfluidic EBG sensor as chemical sensor.

The proposed sensor is compared with recently published microfluidic sensors that operate according to the phase-shift method [31,32,33,34]. The parameters for these sensors are summarized in Table 3, where *f_opr_* denotes sensor operating frequency and *Δφ_max_* is the maximal phase shift. Table 3 also shows specific application of the compared sensors and used fabrication technologies.

The proposed sensor has comparable characteristics and its fabrication process is the simplest and the cheapest compared to other published sensors. Although one may argue that the sensor published in [34] has the largest phase shift, it should be noted that this sensor requires the most demanding fabrication process. Compared with the sensor proposed in [31], which fabrication complexity is fairly similar, the proposed EBG sensor shows 65% better sensitivity. Therefore, the proposed design represents a good candidate for the design of a high-performance sensor since it reconciles the requirements for good sensitivity, compactness and simple fabrication.

## 6. Conclusions

A novel microfluidic microstrip EBG sensor realized using simple 3D printing process has been proposed in this article. The proposed sensor is composed of the microstrip line, defected EBG structure etched in the ground plane, and microfluidic channel embedded in the microstrip substrate. The EBG structure with single defect placed beneath the channel is used as a pattern in the ground plane to improve sensor sensitivity. The microfluidic channel is fabricated using conventional 3D printing technique without any supporting material simultaneously with the microstrip substrate.

The operating principle of the sensor is based on phase-shift measurement of the propagation signal at single operating frequency. When a fluid flows in the microfluidic channel, the phase of the propagating signal changes due to the different permittivity of the fluid. The sensor dimensions were optimized using electromagnetic simulations while performances of the proposed sensor were validated by measuring the phase response for different fluids in the microchannel. 

The measurement results of the fabricated sensor show that the change of the permittivity of fluids in the microchannel from 1 to 80 results in the phase shift of 86°. Moreover, the proposed sensor shows relatively high and almost linear sensitivity for fluids which dialectic constant is lower than 30. The potential application is demonstrated by the implementation of a proposed sensor for the detection of toluene concentration in toluene–methanol mixture. The experimental results show that the phase difference linearly changes when the concentration of toluene changes from 0% to 100% with sensitivity of 0.54° per percentage of toluene.

In this paper, we propose a novel low-cost, reusable, and easily fabricated design that uses small volumes of fluid. The proposed sensor is characterized with relatively high sensitivity and linearity, which makes it a suitable candidate for monitoring small concentrations of specific fluid in different mixtures. For future consideration, we are planning to design supporting electronics, to minimize the sensor dimensions, and improve its sensitivity. The sensor will be further tested on various fluids and mixtures used in biomedical applications and industry.

## Figures and Tables

**Table 1:** Dielectric properties of analysed fluids at room temperature.

Fluid	*ε_r_*	tan*δ*
Toluene	2.3	0.04
Isopropyl	19.7	0.799
Ethanol	24.5	0.941
Methanol	32	0.659
Water	80.1	0.123

**Table 2:** Calculated, simulated and measured phase shifts for different fluids in the microchannel.

Fluid	*ε_sf_*	*ε_eff_*	*Δφ_cal_*	*Δφ_sim_*	*Δφ_meas_*
Air	2.6946	1.5014	0	0	0
Toluene	2.6987	1.5026	17.02	16.39	17.7
Isopropyl	2.7518	1.5183	48.45	58.05	47.5
Ethanol	2.7657	1.5224	62.13	74.47	55.9
Methanol	2.7868	1.5287	76.12	79.12	71.7
Water	2.9066	1.5641	85.75	84	86.7

**Table 3:** Comparison of the characteristics of the proposed sensor and other recently published sensors that operate according to the phase-shift method.

Ref.	*f_opr_*	*Δφ_max_* [deg]	Fabrication Technologies	Applications
[31]	900 MHz	52 (*ε_r_* = 1–80)	Polymethyl methacrylate (PMMA) + micromachining Coplanar waveguide, CPW (composite right-left handed transmission line, CRLH TL with shorted stub)	General microfluidic
[32]	1.84 GHz	94 (*ε_r_* = 1–80)	CPW with Polydimethylsiloxane (PDMS) microfluidic channel on top of glass wafer Borofloat33	General microfluidic
[33]	1 Hz–10 MHz	90 (*ε_r_* = 1–80)	Silicon-on-insulator (SOI) wafer with Cr/Au electrodes, PDMS fluidic	Red blood cell characterization
[34]	2–10 GHz	136 (at 6 GHz) (*ε_r_* = 1:36)	RF micro-electro-mechanical system (MEMS), chemical vapor deposition (CVD) Parylene, Parylene surface-micromachining, Silicon, Cr/Au electrodes	CWP phase shifter with integrated micropumps
This work	6 GHz	86 (*ε_r_* = 1–80)	3D printing Microstrip	General microfluidic/Toluene sensor
